# The Evolving Role of Radiotherapy in Early Stage Hodgkin’s Lymphoma

**DOI:** 10.4084/MJHID.2014.035

**Published:** 2014-06-01

**Authors:** Umberto Ricardi, Andrea Riccardo Filippi, Cristina Piva, Pierfrancesco Franco

**Affiliations:** Department of Oncology, Radiation Oncology, University of Torino, Torino, Italy

## Abstract

Radiation therapy has a key role in the combined modality treatment of early-stage Hodgkin’s Lymphoma (HL). Nevertheless, late toxicity still remains an issue. A modern approach in HL radiotherapy includes lower doses and smaller fields, together with the implementation of sophisticated and dedicated delivery techniques. Aim of the present review is to discuss the current role of radiotherapy and its potential future developments, with a focus on major clinical trials, technological advances and their repercussion in the clinical management of HL patients.

## Introduction

In the era of modern chemotherapy and new highly effective targeted agents, many clinicians may perceive external beam radiotherapy (RT) as an old-fashioned treatment for Hodgkin’s Lymphoma (HL). In fact, the initial demonstration of X-ray effectiveness in HL was made a century ago,^1^ while the first clinical results on disease control and survival have been published in 1935 and 1950.^2^,^3^ However, we are still using this powerful single agent, albeit in a very different way than in early years. For decades Extended Fields Radiotherapy (EF-RT) has been considered the standard treatment for early stage HL, on the basis of the ground-breaking work published by Kaplan in 1968.^4^ It has later become evident that EFRT was associated with a high risk of treatment-related complications, mainly represented by heart diseases, secondary cancers and endocrine dysfunctions.^5^,^6^,^7^ Concomitantly, chemotherapy has been shown to improve results when combined with radiation in early stages.^8^ A large number of subsequent randomized controlled trials, designed and conducted over the last 20 years, lead to re-think the role of RT, modifying its indications and use and questioning its incorporation in such combinations because of concerns about late toxicity. The technological “revolution,” occurred over the last 15 years in Radiation Oncology, made also possible a different technical approach to HL, by applying the new concepts of high-precision image-guided and intensity-modulated RT, even when doses in the range of 20–30 Gy were delivering.

Aim of this review is:

- to summarize and discuss the main changes and the current role of RT in the treatment for HL, and
- to delineate the present and future research paths in RT, focused on maintaining efficacy while minimizing late effects on long-term survivors.

## Overview of Clinical Trials

The initial use of RT was based upon extensive treatment volumes covering both involved and uninvolved lymphatic sites. For the most common presentations in early stages, for example, neck and mediastinum, this approach consisted of subtotal nodal irradiation (STNI), to the dose of 40–44 Gy. The results obtained in the time lapse 1962–1984 by the Stanford group in early stages with EFRT show complete remission rates of 100% and recurrence-free survival (RFS) rates of 80% in stages IA, IIA and IIB without large mediastinal tumors.^9^ In the eighties (1988–1994), the German Hodgkin Study Group (GHSG) designed the HD4 trial, one of first studies to address a specific RT-related question. The major aim of HD4 was to show whether the radiation dose to the non-involved lymphatic regions could be reduced while maintaining an effective tumor control. Patients with early stage HL without risk factors (large mediastinal mass, extra-nodal extension, massive spleen involvement, > 3 lymph node areas, high Erythrocyte Sedimentation Rate) were randomized between 40 Gy EF-RT (arm A) and 30 Gy EF-RT plus additional 10 Gy to the Involved Field (IF) region (arm B). Results showed no statistically significant differences in RFS and overall survival (OS) between the 2 treatment arms, but the overall recurrence rate approached 20%. As relapsing patients underwent an effective salvage therapy, RFS after 7 years came up to 80%, with an overall survival rate of 93%.^10^ For this study, GHSG promoted the creation of a task force for quality assurance (QA). For all patients enrolled in the study, a treatment plan was given by the radiotherapy reference Centre based on the documentation of the disease extension on case report forms. After completion of EF-RT, an expert panel analyzed simulation and verification films of every individual patient, as well as treatment data. This retrospective quality control study showed that deviations of radiation treatment portals and radiation doses from prospective treatment prescriptions were unfavorable prognostic factors.^11^ Second generation of trials compared, both in favorable and unfavorable presentations, EFRT vs. IFRT in combination with chemotherapy. Very valuable data came from these studies, which completely changed the previous treatment paradigm, by showing that the combination of systemic agents and RT was superior to EFRT alone, both in terms of disease control and inferior toxicity. Moreover, these trials demonstrated that, when combined with chemotherapy, RT could be safely reduced to the IF region.^12^,^13^,^14^ This evolution also led to an initial important reduction of late toxicity, as described by the 2005 Cochrane review focused on the therapy of early stage HL and second cancer risks.^15^ At the end of the nineties, a decisive step towards a further reduction of the therapeutic burden was made by GHSG in 2 key studies, the HD10 ad HD11 (1998–2002). In these trials, irradiation was performed as IF-RT only in all treatment arms, with reduced total doses in combination with different chemotherapy schedules. The whole treatment strategy was based upon a proper selection of patients by known prognostic factors. In HD10, stage I–II patients without risk factors (no bulky disease, less than 3 involved sites, low ESR values) were randomized in a four-arm study between an IF-RT dose of 30 Gy vs. 20 Gy and 2 vs. 4 cycles of ABVD. Meanwhile, an extensive quality assurance program has been made in order to ensure that IF-RT was performed exactly according to the RT-prescriptions of the protocol.

Results of HD10 were published in 2010:^16^ the 2 chemotherapy regimens did not differ significantly with respect to freedom from treatment failure (FFTF) (p=0.39) or OS (P=0.61). At 5 years, the rates of FFTF were 93.0% (95% confidence interval [CI], 90.5 to 94.8) with the four-cycle ABVD regimen and 91.1% (95% CI, 88.3 to 93.2) with the two-cycle regimen. When the effects of 20-Gy and 30-Gy doses of radiation therapy were compared, there were also no significant differences in FFTF or OS (p=0.61). HD10 showed that the treatment with two cycles of ABVD followed by 20 Gy of IF-RT was equally effective, and less toxic (acute toxicity), compared to treatment with 4 cycles of ABVD followed by 30 Gy IF-RT. Therefore, 2 ABVD cycles plus IFRT 20 Gy emerged as the standard treatment worldwide for low risk patients. The GHSG HD11 trial,^17^ in patients with unfavorable early stage disease presentation (bulky disease, multiple involved sites, high ESR values), showed that, after 4 cycles of BEACOPP, IF-RT 20 Gy was not inferior to 30 Gy, whereas inferiority of 20 Gy cannot be excluded after 4 cycles of ABVD.

At the same time, other research groups tested a chemotherapy alone strategy in early stage HL, based on similar criteria for patients’ selection (low risk of treatment failure). Some of these studies were conducted on children and/or young adults. The CCG 5942 trial showed inferior 10-year event-free survival for the no RT versus the RT arm (82.9% vs. 91.2%, p=0.004). After stratification for risk factors, a significant difference was evident for the low risk patients (89.1% vs. 100%, P=0.001), but not for the intermediate and high-risk groups (78.0% vs. 84% and 79.9% vs. 88.5%, respectively).^18^ Conversely, the GPOH-HD95 trial showed that the omission of RT was safe only for low-risk patients with complete response after chemotherapy (PFS of 96.8% versus 93.6%, p=0.42), whereas this strategy was not proven to be safe for the intermediate and the high risk groups (PFS 69.1% vs. 92.4%, p<0.001 and 82.3% vs. 90.7%, p=0.08, respectively).^19^ In adults, the largest study to compare chemotherapy alone with combined modality therapy was the intergroup HD.6 study (NCIC), designed with the aim of comparing chemotherapy alone (4–6 ABVD cycles) to RT only or with 2 ABVD cycles (according to risk groups), with subtotal nodal irradiation 35 Gy.^20^ An obvious critical point is that STNI is no more part of current treatments protocols, and thus a direct comparison on late toxicity versus chemotherapy alone is unbalanced. In 2010, Herbst et al published a systematic review with meta-analysis of randomized controlled trials comparing chemotherapy alone with CMT in patients with early stage Hodgkin’s lymphoma with respect to response rate, tumor control and overall survival. Five randomized controlled trials involving 1,245 patients were included. The hazard ratio was 0.41 for tumor control and 0.40 for OS for patients receiving CMT compared to chemotherapy alone.^21^

The results of these studies raised an important debate in the scientific community, still ongoing at present. An individual patient meta-analysis was recently undertaken to compare HD10 and HD11 results with HD.6 study. On 406 patients who fulfilled the eligibility criteria, combined modality therapy was shown to give better time to progression (HR=0.44); PFS was superior but without reaching statistical significance, and overall survival superimposable. Remarkably, the difference between the two treatments was particularly evident among patients in partial remission after chemotherapy.^22^

The following logical step was to try to better select patients at lower/higher risk of relapse, and consequently to better adapt the use of consolidation RT. FDG-PET emerged as a powerful tool to predict early chemo-sensitivity in advanced stages,^23^ and was consequently introduced in early stages to stratify patients with different response to chemotherapy. In these studies, functional imaging was used to modulate therapy, comparing chemotherapy alone strategy to combined modality treatment, consisting of a brief chemotherapy followed by low-dose IF-RT, in patients achieving complete remission at FDG-PET. Three major trials were designed over the last years according to this principle, the H10 trial (EORTC/GELA/FIL), the GHSG HD16 trial and the UK NCRI RAPID trial. In all studies, a panel of expert Nuclear Medicine physicians reviewed FDG-PET imaging results. H10 compared ABVD + RT vs. an experimental arm where the treatment was driven by interim (after 2 ABVD cycles) FDG-PET results. Notably, H10 represented a very innovative step for radiotherapy, introducing the new concept of “Involved Node Radiotherapy” (IN-RT), a further reduction of radiation volumes on the basis of pre- and post-chemotherapy imaging.^24^ Patients with favorable presentations according to EORTC criteria were randomized to ABVD × 3 + IN-RT 30 Gy vs. ABVD × 2 and, if PET negative, 2 more ABVD cycles (chemotherapy alone). This trial is now closed, and the final results will be available within next 2 years. An independent data monitoring committee advised to stop the chemotherapy alone arm due to an excess number of relapses (in both favorable and unfavorable arms).^25^ This decision was deeply discussed, as probably a difference in failure-free survival between the 2 arms (the primary endpoint for non-inferiority), was to be accounted in the statistical design at the beginning, even in patients in metabolic complete response. Overall Survival is expected to be the same for both arms after adequate salvage therapy. The ongoing GHSG HD16 trial has more “contemporary” design with regards to RT doses and compares, in favorable patients (according to GHSG criteria), a standard arm consisting of 2 ABVD cycles followed by 20 Gy IF-RT to a PET-guided experimental arm consisting of 2 ABVD and observation (if negative) or IF-RT 20 Gy (if positive). The purely RT-related question on the potential equivalence of IF-RT and IN-RT is being investigated in a parallel trial, the GHSG HD17.^26^

In UK NCRI RAPID trial, low-risk patients with a PET negative finding after 3 ABVD cycles were randomized either to 30 Gy IF-RT or to observation only. Patients with a positive PET were treated with one more ABVD cycle plus 30 Gy IF-RT. Preliminary findings were disclosed firstly at the 2012 ASH meeting^27^ and then, in updated version, at the ISHL 2013 meeting in Cologne.^28^ The number of events needed to complete the statistical analysis is not reached yet, but results suggest, as expected, slightly inferior RFS for chemotherapy alone in comparison with chemo-radiotherapy in PET negative patients, representing 75% of patients using a prudential cut-off for positivity at Deauville’s score 3 (3-year PFS: 90.8% vs. 94.5%, per protocol). PET positive patients had 86.2% PFS rate. OS was equivalent, with most relapsing patients receiving efficient salvage therapies (not always including ASCT). Table 1 summarizes the results of major clinical trials with radiotherapy-related endpoints in early stage HL.

The impact of such studies on the current role of RT outside clinical trials is difficult to evaluate; however, data suggest that the omission of RT, even in selected patients, may lead to inferior relapse-free survival rates. On the other side, the entity of the difference is small and overall survival rates are probably similar. Nevertheless, the use of early PET findings to guide therapy outside clinical trials is generally considered not appropriate, for two main reasons: an unclear role as a prognostic marker in early stage in comparison with advanced stages, with controversial retrospective findings,^29^,^30^ and the need to have a strict quality control on images interpretation in daily clinical routine (in all trials, PET images were centrally reviewed by a panel of nuclear medicine experts).

## Innovations in Radiotherapy and Strategies to Minimize Radiation-Induced Late Toxicity

During the time interval when most of the aforementioned clinical studies were designed and conducted, the world of radiation oncology deeply changed. The transition from EF-RT to IF-RT was relatively easy since IF were “sub-volumes” of EF, and the fields delineation was based on the anatomical boundaries typical of 2D RT, as exemplified by J. Yahalom and P. Mauch in their 2002 classic article.^31^ When CT simulation and 3D reconstruction software became available, radiation oncologists began to delineate smaller involved fields volumes, corresponding to a new way of considering IF-RT in comparison with the 2D era. At the same time, pre-chemotherapy imaging (CT and CT-PET) became the basis for radiotherapy volumes delineation, actually corresponding to involved sites at diagnosis. This concept has been recently defined as “involved-site radiotherapy” (ISRT), according to the HL radiotherapy guidelines, published by the International Lymphoma Radiation Oncology Group (ILROG),^32^ and was developed on the basis of the INRT concept defined by EORTC in H10 trial.^24^ In both INRT and ISRT, the pre-chemotherapy involvement determines the clinical target volume, and the resulting irradiated volume is significantly smaller than with IFRT. When pre-chemotherapy imaging is available, the contouring process could be divided into 4 steps: 1. delineation of the initially involved lymphoma volume on pre-chemotherapy CT (GTV-CT) as determined by morphology; 2. delineation of the initially involved lymphoma volume on pre-chemotherapy PET/CT (GTV-PET) as determined by FDG uptake; 3. pre-chemotherapy PET/CT images co-registration with post-chemotherapy planning CT scan (the GTV-CT and GTV-PET are imported from the pre-chemotherapy CT to the post-chemotherapy CT); 4. delineation of the post-chemotherapy volume using the information from both pre-chemotherapy PET and pre-chemotherapy CT, taking into account tumor shrinkage and other anatomic changes. In this way, a CTV is obtained encompassing all the initial lymphoma volume while sparing normal tissues that were never involved such as lungs, chest wall, muscles and mediastinal structures. INRT actually represents a special form of ISRT, in which pre-chemotherapy imaging is ideal for post-chemotherapy treatment planning. Outside clinical trials specifically investigating new radiation volumes (i.e. H10 or HD17), radiation fields currently used in clinical routine (henceforth to be called IS-RT) are significantly different from the traditional approach of IF-RT. High-quality retrospective clinical data show that INRT is safe and effective in terms of disease control.^33^–^35^

**Figure 1:**
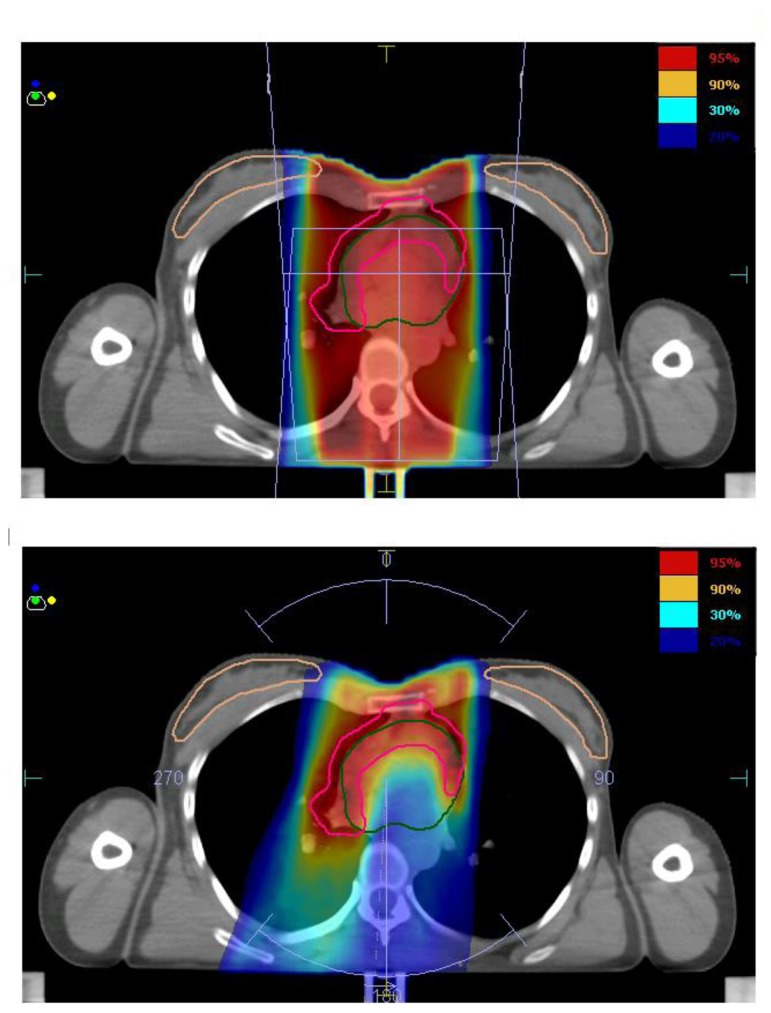
Involved-site 3D-Conformal Radiotherapy (on the top) vs. Intensity-Modulated Radiotherapy (on the bottom) in a patient presenting with Stage IIA mediastinal-supraclavicular Hodgkin’s Lymphoma (Radiation Oncology Department, University of Torino).

Beyond the IS-RT/IN-RT concept, the technological break-troughs in radiation oncology also led to the introduction in clinical practice of highly conformal techniques such as Intensity Modulated Radiotherapy (IMRT). Standard radiation technique consisted in the past of simple parallel-opposed anterior-posterior fields (AP-PA); also in the era of 3D-conformal radiation therapy, the AP-PA approach still represented the most classical solution. Reduced and better defined radiation volumes, together with the advances in treatment planning tools, now allow for the utilization of more conformal radiation therapy, based on more consistent imaging and advanced radiation delivery techniques. As underlined in the ILROG guidelines,^32^ although the advantages of IMRT include the tightly conformal doses and steep gradient next to normal tissues, target definition and treatment delivery verification need even more attention than with conventional RT to avoid the risk of geographic miss and subsequent decrease in tumor control. Image guidance may be required to ensure full coverage during the whole treatment; preliminary retrospective clinical data on the combination of image guidance and IMRT with reduced volumes (ISRT) support the safety of this approach.^36^ Comparative planning studies showed both that INRT may offer a substantial dosimetric benefit in comparison with IFRT and that IMRT may result in a better dose distribution around the target volumes, especially in unfavourable mediastinal presentations (bulky disease, involvement of the anterior mediastinum).^37^–^42^ IMRT can also reduce the mean dose received by critical thoracic structures such as heart and coronary arteries. Figure 1 illustrates an example of the dose distribution achievable with IMRT, in comparison with 3D-CRT, in a mediastinal presentation. The dosimetric gain on healthy tissues achievable with IMRT is usually associated with a larger amount of normal tissues (for example breasts or lungs) receiving very low doses (1–2 Gy out of 30 Gy), with a potential negative impact on radiation-induced secondary malignancies risk. Historically, the shrinkage of radiation fields from EF-RT to IF-RT has been shown to decrease the risk of second cancers, as reported by De Bruin et al.^43^ This effect might be significant also when shifting from IF-RT to ISRT/IN-RT, especially in specific disease presentations (according to the disease extent and the involved lymph nodes anatomical location). Few interesting modeling studies were conducted with the aim of evaluating both the impact of reduced volumes and IMRT on secondary cancers risk in early stage HL.^44^–^47^ Results showed that INRT, at least theoretically, reduces the risk of secondary cancers in comparison with IFRT; the findings on IMRT vs. 3D-CRT were rather unclear, depending on both the IMRT technique and the radiobiological models used for risk estimation. Valuable clinical data on the incidence of secondary tumors after combined modality therapy with INRT-IMRT will only become available over the next years. Table 2 illustrates the time trend in radiotherapy volumes/dose/technology evolution since 1960 to present.

## Conclusions

Early stage HL patients should be possibly included in clinical trials investigating for treatment optimization. In clinical routine, combined modality therapy still represents the standard, with radiation oncologists now having the opportunity to minimize the risks of late toxicity by using a large armada of technological improvements. Long-term follow-up is needed to clarify the clinical impact of these technical advancements on late morbidity.

## Figures and Tables

**Table 1:** Summary of clinical trials investigating for radiotherapy-related endpoints.

Study	N	Median follow-up, mo	Treatment	OS, %	*P*	FFTF, EFS, FFP, PFS, %	*P*
**GHSG (HD4)**^10^	376	86	EFRT 40 Gy	91 at 7 yr	NS	78 at 7 yr	NS
			EFRT 30 Gy	96 at 7 yr		83 at 7 yr	
	
**Istituto Nazionale Tumori**^12^	136	116	ABVD × 4 + STNI	96 at 12 yr	NS	93 at 12 yr	NS
			ABVD × 4 + IFRT	94 at 12 yr		94 at 12 yr	
	
**GHSG (HD8)**^13^	1064	54	COPP/ABVD × 2 + EFRT	90.8 at 5 yr	NS	85.8 at 5 yr	NS
			COPP/ABVD × 2 + IFRT	92.4 at 5 yr		84.2 at 5 yr	
	
**EORTC (H8)**^14^							
Favorable	542	92	STNI	92 at 10 yr	.001	74 at 5 yr	< .001
			MOPP-ABV × 3 + IFRT	97 at 10 yr		98 at 5 yr	
Unfavorable	996		MOPP-ABV × 6 + IFRT	88 at 10 yr	NS	84 at 5 yr	NS
			MOPP-ABV × 4 + IFRT	85 at 10 yr		88 at 5 yr	
			MOPP-ABV × 4 + STNI	84 at 10 yr		87 at 5 yr	
	
**GHSG (HD10)**^16^	1370	90	ABVD × 2 + IFRT 20 Gy	95 at 8 yr	NS	86 at 8 yr	NS
			ABVD × 2 + IFRT 30 Gy	94 at 8 yr		86 at 8 yr	
			ABVD × 4 + IFRT 20 Gy	95 at 8 yr		90 at 8 yr	
			ABVD × 4 + IFRT 30 Gy	94 at 8 yr		87 at 8 yr	
	
**GHSG (HD11)**^17^	1395	91	ABVD × 4 + IFRT 20 Gy	94 at 5 yr	NS	81 at 5 yr	.02
			ABVD × 4 + IFRT 30 Gy	94 at 5 yr		85 at 5 yr	
			BEACOPPbase × 4 + IFRT 20 Gy	95 at 5 yr		87 at 5 yr	
			BEACOPPbase × 4 + IFRT 30 Gy	95 at 5 yr		87 at 5 yr	
	
**CCG (5942)**^18^	826	91	COPP/ABVD × 4 or COPP/ABV × 6 or 6 intensified cycles + IFRT	97.1 at 10 yr	.05	91.2 at 10 yr	.004
			COPP/ABVD × 4 or COPP/ABV × 6 or 6 intensified cycles + NFT	95.9 at 10 yr		82.9 at 10 yr	
	
**GPOH (HD95)**^19^	925	120	OPPA/OEPA × 2	98.5 at 10 yr	NS	97.0 at 10 yr	NS
			OPPA/OEPA × 2 + RT if PR	98.7 at 10 yr		92.2 at 10 yr	
			OPPA/OEPA × 2 + COPP × 2	97.7 at 10 yr	NS	68.5 at 10 yr	<.001
			OPPA/OEPA × 2 + COPP × 2 + RT if PR	98.1 at 10 yr		91.4 at 10 yr	
			OPPA/OEPA × 2 + COPP × 4	100 at 10 yr	NS	82.6 at 10 yr	NS
			OPPA/OEPA × 2 + COPP × 4 + RT if PR	95.3 at 10 yr		88.7 at 10 yr	
	
**NCIC/ECOG (HD.6)**^20^	399	50	ABVD × 4–6	96 at 5 yr	NS	87 at 5 yr	.006
			ABVD × 2 + STNI	94 at 5 yr		93 at 5 yr	
	
**EORTC/LYSA/FIL (H10 interim analysis)**^25^							
Favorable	444	13	ABVD × 3 + INRT	/		100 at 1 yr	.017
			ABVD × 4	/		94.3 at 1 yr	
			ABVD × 2 + BEACOPPesc × 2 + INRT				
Unfavorable	693			/		97.28 at 1 yr	.026
			ABVD × 4 + INRT	/		94.7 at 1 yr	
			ABVD × 6				
			ABVD × 2 + BEACOPP × 2 + INRT				
	
**UK NCRI (RAPID interim analysis)**^27^	420	48	ABVD × 3 + NFT	/		90.8 at 3 yr	
	PET neg.		ABVD × 3 + IFRT	/		94.5 at 3 yr	

**Table 2:** Temporal evolution of radiotherapy for early stage Hodgkin’s lymphoma.

RT Fields	Years	Dose (Gy)	Technique	Planning Methods	Machines
EFRT	1960–1990	40–44	2D RT	2D planning	Cobalt Units; first LINACS
IFRT	1995–2005	30–36	3D-CRT	3D Planning	
			Static-IMRT	Forward/Inverse planning	LINAC with Multileaf Collimator
ISRT/INRT	2005–present	20–30	Static IMRT	Inverse Planning	LINAC with Multileaf Collimator
			Arc-therapy	Biologic Optimization	LINAC with Dinamic MLC and Image-Guidance
			Tomotherapy	Multimodality Imaging	Volumetric Modulated Arc Therapy
				Dose Painting	Helical Tomotherapy
				Image-Guided Radiotherapy	
